# Over ninety years old: Does high cognitive reserve still help brain efficiency?

**DOI:** 10.1007/s00426-023-01881-1

**Published:** 2023-10-06

**Authors:** Elisa Carta, Alice Riccardi, Silvia Marinetto, Sara Mattivi, Enrico Selini, Veronica Pucci, Sara Mondini

**Affiliations:** 1 Department of Biomedical Sciences, Multiple Sclerosis Centre Binaghi Hospital, University of Cagliari, Cagliari, Italy; 2 Multiple Sclerosis Centre, Department of Neurosciences-DNS, University-Hospital of Padua, Padua, Italy; 3 Independent Researcher, Padua, Italy; 4 Dipartimento di Filosofia Sociologia Pedagogia e Psicologia Applicata (FISPPA), University of Padua, Padua, Italy; 5 Human Inspired Technology Research-Centre, University of Padua, Padua, Italy; 6 Servizi Clinici Universitari Psicologici (SCUP), University of Padua, Padua, Italy; 7 Present Address: Independent Researcher, Padua, Italy

## Abstract

Nonagenarians and centenarians, also called oldest-old, are a very heterogeneous population that counts a limited number of individuals as it is a real challenge to reach this goal. Even if it is well known that cognitive reserve can be considered a factor in maintaining good cognitive functioning in ageing, only very few studies have been carried out on the role of cognitive reserve (CR) in the oldest-old people. The aim of this study is to investigate the relationship between cognitive reserve and cognitive functioning in a population living in a specific region of Italy, the Blue Zone in Sardinia. This population is characterised by extreme longevity and distinctive historical, geographic, social, linguistic and nutritional features. The cognitive Reserve Index questionnaire (CRIq) and the short cognitive Esame Neuropsicologico Breve-2 (ENB-2, Brief Neuropsychological Examination) were administered to 67 participants, all aged between 90 and 105 years old. The CRIq was a predictor of neuropsychological performance for the global score of the battery of tests, ENB-2 (ENB-tot) and also for 7 out of 16 of its sub-tests. All except one (Token) tapped executive functions (Interference memory at 10 and 30 s, TMT-B, Overlapping figures, Abstraction, Fluency). Results highlight that also in the oldest-old population CR has a positive effect on cognition, especially on executive functioning.

## Introduction

Ageing is a universal and multifactorial process characterised by progressive development, maturation and decline of the organism from birth to death. It is denoted by a gradual and progressive loss of functional capacity and increased comorbidity, both directly proportional to age (Paolisso & Boccardi, 2014).

Age represents an important risk factor for dementia onset as do low education, poor physical activity and vascular factors (Pierce et al., 2017). Even though the loss of functional and cognitive abilities is physiological, it remains essential to distinguish between normal and pathological ageing and to identify factors which play a role in this time of life (Pierce et al., 2017).

Only very few studies have investigated non-pathological cognitive functioning in the oldest-old (defined as aged over 90, World Health Organization, 2017). In this population, the cognitive profile is characterised by a general decline, particularly in memory, attention and executive functions (Salthouse, 1998).

In the context of ageing, cognitive/brain reserve can be a protective factor against decline (Lavrencic et al., 2018; Legdeur et al., 2019). The model of brain and cognitive reserve (Satz, 1993; Stern, 2002) states that the brain actively contrasts cerebral damage using other cognitive processes or by implementing compensatory mechanisms.

Many factors may contribute to brain reserve: cerebral dimensions, amount of neurons and synapses, brain-derived neurotrophic factors (Chicherio et al., 2012; Lipsky et al., 2007), and different factors contribute to cognitive reserve (CR): lifestyle, job, education, life experiences, leisure activities and physical activity (Le Carret et al., 2003; Mondini et al., 2022; Pool et al., 2016; Pucci et al., 2023). Bilingualism may also be involved: continuous switching between two (or more) different languages requires the use of different neural networks and high cognitive engagement (Calvo et al., 2016; Craik et al., 2010).

Another important concept is brain maintenance (Cabeza et al., 2018; Nyberg et al., 2012; Steffener et al., 2016; Stern et al., 2020), which refers to the relative absence of change in neural resources over time which preserves cognition in ageing. Brain maintenance and CR support each other in a complementary way. The former indicates preservation of the brain, while the latter refers to sustaining cognition that contrasts brain changes. These two mechanisms can be modulated by genetic and/or environmental factors during the lifespan (see also the Consensus Conference on Reserve and Resilience at https://reserveandresilience.com/).

In Italy, the number of people over 65 years old is constantly growing, and it is expected to reach 35% of the population around 2045–2050 (Istat, 2021). At the same time, life expectancy is increasing (5 years or more in 2070) and the number of people who will reach the age of 90 or more will very likely increase, too. It seems therefore fitting to study the relationship between CR and cognitive performance in the oldest-old population. In recent years, some communities around the world have been discovered where survival into very old age is not episodic but occurs in a significant part of the population. One of these populations lives in the Blue Zone, on the Mediterranean island of Sardinia. A lot of interest surrounds this area and its population because it could be good ground to study what makes individuals live longer and stay cognitively and physically healthy (Pes et al., 2020). Many hypotheses have been proposed focusing on different lifestyle factors. For example, Herbert et al. (2022) highlighted the importance of the physical activity of centenarians of this zone. Daily moving other than hobbies, and work beyond the expected age of retirement, would have a key role in their wellbeing.

The study of Ruiu et al. (2022) suggests, instead, that the socio-cultural context of the Blue Zone promotes great autonomy in facing daily life in older adults, and encourages their active role in the community passing on local traditions and cultural values to younger people. In consequence, depression symptoms are significantly reduced compared to people from other rural areas of Sardinia. Nieddu et al. (2020) compared two very long-lived populations: one living in Costa Rica (Nicoya peninsula) and the other in the Sardinia Blue Zone. They found that a particular plant-based diet was shared by the two populations and concluded that this could be at the basis of their optimal state of health and long-lasting functional and physical capacity. A high-functioning brain in older age is another factor which accompanies active life and which is promoted by cognitive reserve, as shown by many studies in older adults (e.g., Cabeza et al., 2018).

In this study, we would like to go further and investigate the oldest-old individuals living in Sardinia as a reference population in order to explore the relationship between their CR and their cognitive performance.

The study was approved by the Ethical Committee of the School of Psychology, University of Padua (*N = *4857) and it was conducted in accordance with the Declaration of Helsinki.

## Materials and methods

Sixty-seven participants (47 women and 20 men) aged between 90 and 105 (*M = *93.66; SD = 3.23) were enrolled in this study. Mean education was 4.69 years (SD = 2.88), ranging from 0 to 18. All participants were born and resided in Sardinia, mainly in the province of Nuoro. They were all living at home and were autonomous in their daily life: twenty of them lived alone, and the others lived with their partners or with a son/daughter. Participants with neurological issues or recent hospitalizations at the time of assessment were excluded from the sample. In general, the overall education of the sample was very low: 25 people had attended primary school with only 12 of them completing the 5-year course. Only two people had a university degree and one had never attended school, although she had learnt to read and write by herself. This low level of formal education reflects the social and cultural context of the 1920s/1930s in Sardinia.

During data collection, seven individuals were still working: four shepherds, aged 91, 91, 96 and 97, a priest aged 91, a florist aged 91 and a shopkeeper aged 101. All participants were bilingual because they used both the Sardinian and Italian languages every day. Many of them spent their free time doing various activities such as reading, needlework, poetry, gardening, and travelling. Some of them even used social networks.

Two tools were used in the present study: (1) the Brief Neuropsychological Examination 2 (Esame Neuropsicologico Breve 2—ENB 2; Mondini et al., 2011), made up of 16 tests to measure different cognitive functions and (2) the Cognitive Reserve Index questionnaire (CRIq, Nucci et al., 2012) to quantify participants’ Cognitive Reserve.

ENB-2 is a neuropsychological battery often used in Italy with older adults (see, for example, Zangrossi et al., 2021). It includes sixteen tests to assess different cognitive functions: (1) *Digit span* (repetition of series of digits) for short-term memory; (2) *Immediate* and (3) *Delayed recall prose memory* (repetition of a short story at two different delays), to evaluate long-term memory; (4) *Interference memory at 10 s* and (5) *Interference memory at 30 s* (memorizing 3 letters while counting by two) to evaluate capacity to perform a double-task and divided attention; (6) *Trial making test, part A* (linking numbers in ascendent order with a line on a sheet of paper), to evaluate selective attention and (7) *Trial making test, part B* (alternating linking with a line numbers, in ascendent order, and letters, following the alphabet, on a sheet of paper), to evaluate selective and switching attention; (8) *Token test* (executing simple and precise verbal orders), to evaluate verbal comprehension; (9) *Word phonemic fluency test* (producing words beginning with the same letter), to evaluate access to lexicon and executive functions in strategy of searching; (10) *Abstract reasoning test* (finding the semantic category shared between two concepts), to evaluate abstract thinking and reasoning; (11) *Cognitive estimation test* (finding the closest answer to unknown and unusual questions), to evaluate capacity of estimation and executive functions in finding solutions; (12) *Test of overlapping figures* (recognition of individual shapes within a complex image with overlapping meaningful figures), to evaluate visual perception and executive functions in selecting different figures from a background; (13) *Spontaneous drawing test* (drawing a daisy with a stem and one leaf), to evaluate imagery and drawing abilities; (14) *Copy drawing test* (copying the drawing of a house), to evaluate praxic and visuo-constructional abilities; (15) *Clock drawing test* (drawing a clock with all the numbers and placing the hands at a specific time), to evaluate imagery, planning and visual constructional abilities*;* and (16) *Praxis test* (executing meaningful gestures or copying meaningless gestures with hands and arms), to evaluate praxic abilities, that is, the capacity to use parts of the body to make specific gestures.

The battery provides both a score for each single cognitive task and a total score (Global cognitive index) that indicates the overall cognitive profile. The normative sample from 702 individuals of different age, sex and education provides specific cutoff scores for individuals over 80 years old. The 5th percentile was used to determine normality cutoff scores and all performances below this cut-off are considered potentially impaired. The battery shows good psychometric characteristics with good discriminant validity in identifying clinical groups, and good test–retest reliability (from 0.57 to 0.97) (Mondini et al., 2011).

The CRIq is a semi-structured interview to evaluate a cognitive reserve and consists of three sections: the first relates to education (CRI-Education); the second asks about occupations (CRI-Working Activity); and the third collects information about leisure activities (CRI-Leisure Time). Three linear models are used to reduce the age effect on the scores of the three CRIq sections which are the residuals of the relative linear models, standardised and transposed to a scale with *M = *100 and SD = 15. Thus, each participant is compared with the corresponding age class. CRI total is the average of the three sub-scores, again standardised and transposed to a scale with *M = *100 and SD = 15. CRI is classified into five levels: low (< 70), medium–low (70–84), medium (85–114), medium–high (115–130) and High (> 130). In this study, we administered the digital version of the questionnaire (http://www.cognitivereserveindex.org/calcolo/calcolo.html).

The recruitment was carried out by accessing municipal registers. People were contacted and asked to participate voluntarily in the study. After the procedure was explained to the participants their written informed consent was obtained.

The neuropsychological assessment, CRIq (Nucci et al., 2012) and the ENB-2 tests (Mondini et al., 2011), was carried out in one session at participants’ home and it lasted approximately 1 h and a half. Participants often felt more competent in speaking Sardinian than Italian; therefore, answers in both languages were accepted.

## Results

Data collected were analysed using R Studio (version 4.1.0, RStudio Team, 2020) and Jamovi (version 1.6, The Jamovi Project, 2021).

Participants’ Cognitive Reserve Index (CRI) was quite heterogeneous, ranging from 88 to 165 (*M = *111, SD = 16.7), as already reported for education which ranged from 0 to 18. Descriptive statistics of participants’ demographic features and their performance in every single task of ENB-2 and global score (ENB-2 tot) are reported in Table 1.

**Table 1:** Descriptive statistics of the sample demographic features (age, education and CRI) and of all scores obtained in all ENB-2 tasks and ENB-2 total score

	Mean	Median	Mode	SD	Min	Max	Skewness	Kurtosis
Age	93.66	92	92	3.2	90	105	1.5	2.4
Education	4.69	5	5	2.9	0	18	2.2	7.9
CRI	115.7	111	104	16.7	88	165	2.1	6.8
Digit span	4.5	4	4	0.8	3	7	0.8	0.3
Immediate memory	6.8	7	6	2.4	3	15	0.9	1.6
Delayed memory	8	7	5	4	0	20	0.8	0.7
Interference memory (10 s)	3	2	2	2.2	0	9	0.8	− 0.1
Interference memory (30 s)	2.5	2	2	2	0	8	0.6	− 0.3
TMT-A	277.2	183	999	282.1	34	999	2	2.6
TMT-B	329.7	296	121	166.3	121	708	0.9	0.2
Token	4.6	4.5	5	0.5	3	5	− 1.2	0.6
Fluency	6.4	5.6	3.6	3.2	1.6	17	1.3	1.9
Abstraction	3.8	4	4	1.6	1	6	− 0.2	− 0.9
Overlapped figures	14.6	14	14	6.5	0	33	0.5	0.4
Cognitive estimation	3.8	3	3	6.5	1	5	− 0.2	− 0.4
Copy drawing	0.8	1	1	0.7	0	2	0.3	− 1.1
Spontaneous drawing	1.2	1	1	0.8	0	2	− 0.4	− 1.1
Clock	4.8	4	4	2.5	0	9.5	− 0.1	− 0.5
Praxis	5.8	6	6	0.5	3	6	− 3.6	14.4
ENB-2 tot	47.9	47	43	9.7	28	69	0.2	− 0.3

Regression analyses were carried out to verify the predictive value of CRI for participants’ cognitive performance at ENB-2 in every single task and as global performance (ENB-2 tot). Age was not considered a covariate because of its very low variability (range 90–105) and to avoid multi-collinearity since its high correlation with CRI. Education was also not considered as co-variate in order to avoid collinearity, as CR includes years of education.

A statistically significant effect of CRI was found for the ENB-2 total score (*R*^2^ = 0.182, *p* < 0.001), and for 7 out of 16 single tasks: Interference memory at 10 s (*R*^2^ = 0.145, *p = *0.015), Interference memory at 30 s (*R*^2^ = 0.072, *p = *0.028), TMT-B (*R*^2^ = 0.223, *p = *0.047), Token (*R*^2^ = 0.081, *p = *0.019), Fluency (*R*^2^ = 0.323, *p* < 0.001), Abstraction (*R*^2^ = 0.092, *p = *0.012) and Overlapped figures (*R*^2^ = 0.132, *p = *0.002). Table 2 summarises the regression models used.

**Table 2:** Details of regression models having ENB-2 tasks as dependent variables and CRI as independent variable

	Model fit measures		Model coefficients	
			Intercept	CRI
Tasks	*R*^2^	*p*	Estimate	Estimate
Digit span	0.052	0.063	3.225	0.011
Immediate memory	0.032	0.149	3.844	0.026
Delayed memory	0.019	0.270	4.222	0.033
Interference memory (10 s)	0.145	** < 0.001**	− 2.773	0.050
Interference memory (30 s)	0.072	**0.028**	− 1.159	0.032
TMT-A	0.022	0.228	568.110	− 2.515
TMT-B	0.223	**0.048**	809.684	− 3.854
Token	0.081	**0.019**	3.538	0.009
Fluency	0.323	** < 0.001**	− 6.261	0.110
Verbal abstraction	0.092	**0.012**	0.348	0.030
Overlapped figures	0.132	**0.002**	− 1.641	0.141
Cognitive estimation	0.036	0.122	1.947	0.013
Copy drawing	0.003	0.683	0.590	0.002
Spontaneous drawing	0.033	0.141	0.267	0.008
Clock	0.019	0.267	2.507	0.020
Praxis	< 0.001	0.855	5.739	< 0.001
ENB-2 tot	0.182	** < 0.001**	19.388	0.247

Significant models are reported in bold

## Discussion

The purpose of this study was to explore the relationship between cognitive reserve (CR) and neuropsychological performance in nonagenarians and centenarians who are living in a specific area of Sardinia, the Blue Zone. Our interest was to understand whether cognitive reserve can predict neuropsychological performance in oldest-old individuals. Our analysis shows that high cognitive reserve predicts better cognitive efficacy, especially in executive function tasks, and this positive relationship is found in a sample of oldest-old individuals. Participants generally showed a mean ENB-2 total score well above the cut-off for over 80. However, as the normative data of ENB-2 do not adequately represent their age range, ENB-2 may even have underestimated their performance.

Furthermore, considering the single tests of ENB-2, regression analyses show that CR predicts better performance on several individual tests such as Token, Interference memory (10 and 30 s), TMT-B, Fluency, Abstraction and Overlapping figures. With the exception of the Token test, all these tasks involve executive function. The link between CR and executive function seems to be confirmed by other data in the literature. For example, Oosterman et al. (2021) (but see also Siedlecki et al., 2009; Satz et al., 2011; Colangeli et al., 2016; Roldán-Tapia et al., 2012) showed that CR attenuates the effect of age on executive functioning in old to very old adults. As executive functions are core mechanisms of cognition, they may represent the compensatory mechanisms of CR (Tucker & Stern, 2011).

Another interesting point is that, although this population has very low average education and low cognitive demanding working activities, their CR is at a medium–high level due to stimulating leisure activities. Some participants, for example, knew by heart a few of the great classics of literature, such as The Divine Comedy and The Iliad, and took part in poetry competitions. Others organised and led hunting parties, or went on holiday to thermal areas, or went on pilgrimages. According to previous studies (e.g., Lee et al., 2020), cognitively challenging leisure activities play a fundamental role in the construction of cognitive reserve, especially in not so well-educated people, and that is the case for our special participants.

The power of cognitive reserve in our population clearly shows how an active lifestyle is beneficial in the elderly and promotes well-preserved ageing. Whatever activity requires engaging, thinking and social collaboration maintains our brain’s well functioning, reduces hospitalisation and self-sufficient independence in daily living. Thus, promoting a cohesive and socially active community could be an excellent strategy to be taken up by our advanced societies.

Notwithstanding the value of our results, the study is not without some limitations. Our sample was fairly small and to verify the generalizability of our data, future research should increase the sample size in order to achieve a large open-access database of the Italian population. A control group should also be included, for example, a group of younger Sardinian elders or a group of oldest-old living in other areas of Italy, to evaluate and compare performances. Future studies should consider these points to understand more in depth the co-occurrence of these variables in determining healthy ageing.

In conclusion, as the literature has underlined, environmental and genetic factors undoubtedly contribute to high brain maintenance and high cognitive performance. On the other hand, we found that cognitive reserve, mainly derived from leisure activities, continues to modulate and strengthen cognitive functioning. We can say that, in addition to brain maintenance, cognitive reserve contributes to the mental health and the general well-being of the oldest-old. Although at this age biological changes strongly impact cognitive functioning, CR still has a role in preserving cognitive health and ability to function successfully.

## Data Availability

The datasets generated during and/or analysed during the current study are available from the corresponding author on reasonable request.
